# Supplementary material for: How can county-level industrial clusters in China promote urban-rural integration?—A study on the configuration effects based on fsQCA
Source: PLoS One. 2025 Sep 30;20(9):e0329972. doi: 10.1371/journal.pone.0329972 (PMC12483206; doi:10.1371/journal.pone.0329972)
Supplement: Attachment — Submitted filename: Response to Reviewer #2.docx [file pone.0329972.s002.docx]

**Reviewer #2**
***Thank you very much for your professional and insightful review comments. Below are the revisions made in response to each of your suggestions:***

For your convenience, more detailed revisions and annotations have been added to the latest version of the PDF manuscript.

1.The paper should be structured as:

Introduction

Literature review

Methodology

Results

Discussion

Conclusion

**Revision:**

**Q1: The section layout has been adjusted according to your suggestion.**

2.The literature review is somewhat thin. Please provide a more detailed review relevant to your research topic, and clearly indicate the shortcomings of existing studies.

3.The theoretical foundation states that county - level industrial clusters promote urban - rural integration through four paths: "industry linkage, technology transfer, employment coordination, and service balance." However, it fails to specify the research gaps. What did prior studies overlook?How does the current study build on or diverge from earlier research? Identifying these gaps strengthens the theoretical basis.

**Revision:**

**Q2-3: Expanded the literature review by adding a discussion of research gaps and clarified the theoretical contributions of this study.**

4.The result variables borrow primary indicators from China's urban development index system and design secondary indicators for urban - rural integration. The validity of using these to measure county - level urban - rural integration is questionable. Clarify the connection between urban - ruralintegration indicators and China's urban development index system.

**Revision:**

**Q4: Improved Table 1 by explaining the theoretical basis for the selection of urban-rural integration indicators and providing detailed descriptions.**

5.Explain the basis for setting the calibration points at 75%, 50%, and 25%.

**Revision:**

**Q5: Provided a detailed explanation for the calibration thresholds used in the fsQCA results (updates reflected in Tables 3 and 4).**

6.Check whether the sentence: "This indicates that the low level of 'industrial linkage' is a necessary condition for low - level green development, meaning that insufficient industrial linkage is a key constraint in regions with low urban - rural integration development." has any issues,especially the appropriateness of "low - level green development" in this context.

**Revision:**

**Q6: Revised this expression and incorporated case analyses related to "low-level urban-rural integration" for better clarification.**

7.The path analysis in Section 4 should be deepened with sample case integration. Add explanations of sample case distribution and provide representative real - world examples for each path.

**Revision:**

**Q7: Added case studies from Chinese counties to illustrate each pathway more concretely.**

8.Elaborate on the research methods in stages for better replicability. Include a discussion section to introduce the latest references, compare them with your study or others in the field, and offer guidance and ideas for future research. Introduce the research questions if applicable.

**Revision:**

**Q8:**

**A: Employed principal component analysis (PCA) and the entropy weight method to measure urban-rural integration indicators (Table 1).**

**B: Clarified the calculation of coupling degree and explained its conceptual application.**

**C: Supplemented the measurement of mechanism variables by introducing the "urban-rural technology transaction coefficient" (Table 2).**

**D: Strengthened the overall validation and analysis of the fsQCA methodology (revisions made across Tables 3, 4, 5, 6, and 7).**

9.The conclusion should include an analysis of the study's limitations and prospects for future research.

**Revision:**

**Q9: Added a discussion of research limitations and outlined potential future research directions in the conclusion.**

***Finally, thank you again for your valuable review comments!***
