# Supplementary material for: How can county-level industrial clusters in China promote urban-rural integration?—A study on the configuration effects based on fsQCA
Source: PLoS One. 2025 Sep 30;20(9):e0329972. doi: 10.1371/journal.pone.0329972 (PMC12483206; doi:10.1371/journal.pone.0329972)
Supplement: Attachment — Submitted filename: Response to Reviewer 3.docx [file pone.0329972.s003.docx]

Response to Reviewer 3 Comments

Dear Reviewer 3,

Thank you for your careful review and detailed suggestions, which have been instrumental in improving the quality and clarity of our manuscript. We have addressed each of your comments as follows:

**Abstract Revision**: The abstract has been thoroughly revised to correct grammatical errors and improve clarity. The updated version more clearly outlines the research background, methodology, key findings, and policy implications.

**Model Formulation and Application**: In the methodology section (4.3), we have added a formal model equation representing the fsQCA framework, along with a detailed explanation of the set-theoretic logic and configuration process. We also elaborated on how the model is implemented in the empirical analysis, including truth table construction, calibration thresholds, and logical minimization steps.

**Improvement Measures**: To respond to your request for practical recommendations, we added a new subsection discussing optimization strategies. These include potential integration with dynamic panel data, incorporation of policy-related variables, and suggestions for multilevel fsQCA applications in future research.

**Terminology Consistency**: We have corrected the inconsistency in the term “urban-rural integration,” particularly in the title of Figure 1, to ensure terminological uniformity throughout the manuscript.

**Language Polishing**: Following your recommendation, the entire manuscript has undergone a comprehensive language refinement process. We revised sentence structures, improved terminology usage, and enhanced the overall readability to meet international academic standards.

We are grateful for your detailed critique, which has significantly strengthened the clarity, coherence, and rigor of our study.

Sincerely,
June 29,2025
